# *PomiR172d*-*PoARR* module regulates the drought response through the reactive oxygen pathway in tree peony

**DOI:** 10.1093/hr/uhaf252

**Published:** 2025-09-17

**Authors:** Bixi Li, Yining Liu, Haiying Liang, Xiaohui Wang, Sitong Wang, Jiajia Shen, Xiaogai Hou, Lili Guo

**Affiliations:** College of Tree Peony, Henan University of Science and Technology, Luoyang, China; College of Tree Peony, Henan University of Science and Technology, Luoyang, China; Department of Genetics and Biochemistry, Clemson University, Clemson, South Carolina, USA; Tree Peony Research Institute, Luoyang Academy of Agriculture and Forestry Sciences, Luoyang, China; College of Tree Peony, Henan University of Science and Technology, Luoyang, China; College of Tree Peony, Henan University of Science and Technology, Luoyang, China; College of Tree Peony, Henan University of Science and Technology, Luoyang, China; College of Tree Peony, Henan University of Science and Technology, Luoyang, China

## Abstract

Tree peony (*Paeonia* section *Moutan* DC.) is a unique group of precious woody species with high ornamental, medicinal, and oil value. A drought environment severely restricts their yield and quality. However, the screening and identification of miRNAs in response to drought stress of tree peony has not been reported. In this study, *Paeonia ostii* ‘Fengdan’ were treated with mild drought, severe drought, and rehydration, respectively. The results of phenotypic observation and physiological characteristics showed that the cell membrane of *P. ostii* leaves was damaged by drought stress and had a self-regulation function. Combined with multi-omics analysis (transcriptomics, miRNA, and degradome), a total of 883 miRNAs with significant differential expression were identified, and the expression regulation networks of miRNAs and target genes were constructed. A set of 19 different miRNAs was found to regulate 189 different genes. Drought-responsive miRNA–mRNA–TF modules like *miR172d-ARR* (Arabidopsis Response Regulator), *miR396g-STAT* (Signal Transducer and Activator of Transcription), and *miR168-DBB* (Double B-Box) were discovered. By cloning the key miRNA *PomiR172d* and its target gene *PoARR* and conducting genetic transformation to verify its function, analyzing the permeability of cell membrane and enzyme activity of ROS in transgenic plants, the molecular regulatory mechanism of the *PomiR172d-PoARR* module of tree peony in response to drought stress was revealed. Our studies lay the foundation for future research on the regulatory mechanism of tree peony in response to drought stress and provide a theoretical basis for the improvement and cultivation of drought-resistant varieties of tree peony.

## Introduction

Tree peony (*Paeonia* section *Moutan* DC.) is a group of woody perennial deciduous shrubs of the section *Moutan*, genus *Paeonia*, and family *Paeoniaceae* [1]. All wild species are unique to China [2]. The flowers are ornamental, the seeds can be extracted for oil, and the roots can be used for medicine. Thus, tree peony is an extremely precious multi-purpose plant with high values [3]. Known as the ‘King of flowers’, tree peony flowers are large and colorful, beautiful and fragrant [4]. It symbolizes wealth and prosperity and is renowned at home and abroad as a symbol of Chinese civilization [5]. Nowadays, cultivated tree peonies have been spread all over the world, and people’s enthusiasm for cultivating and planting peonies is increasing [2].

Drought stress is a significant factor that restricts flowering quality and seed yield of tree peony [6]. In particular, the recent expansion of cultivation in China has mainly concentrated in arid, semi-arid or seasonally arid areas [7]. Also, potted tree peonies, especially during the Chinese Spring Festival, are increasingly favored by people. However, in the cultivation process of potted tree peonies, water management consumes a lot of manpower and material resources, which increases the production and maintenance costs. It is urgent to develop new tree peony varieties of high quality with high drought tolerance and high ornamental value.

Due to its economic significance, there is rapid progress in genomic resources and studies of tree peony. The whole genomes of three cultivars, ‘Luoshen Xiaochun’ [5], ‘Fengdan’, [8] and ‘Dahua Huang’ [9], became available in the last three years, providing a solid foundation for genomics research. The publication of full-length transcriptome sequences provides reliable reference sequences for cloning and functional analysis of tree peony genes [10, 11]. The application of virus-induced gene silencing (VIGS) to verify the function of tree peony genes [12–14] has provided an alternative to the lack of a mature genetic transformation system for homologous transformation in the species. Examples of functional characterization include *PhRING-H2* [15], *PlTDC* [16], *PoDPBT* [17], *PlWRKY41a* [18], *PlR2R3-MYB* [19], and *PsSOC1* [20–22], revealing molecular regulation mechanisms of important genes. For studies of drought stress, publications include evaluation of drought resistance [23, 24], effects of exogenous substances [4, 16, 25–27], SSR loci related to drought tolerance [6, 28], and mining of drought tolerance genes [29–31]. General stress resistance genes that have been studied in tree peony include *TDC* [16], *CCoAOMT* [32], *DHN1* [33], *HSP70* [34], *WRKY65* [35], *R2R3-PsMYB1* [36], and *DHN-YSK2* [37]. Furthermore, functional identification and promoter analysis of *PsMPT* [38] and *DREB2* [39] have been reported.

MicroRNAs (miRNAs) are key players in plant regulatory networks. miRNAs interact with target genes at transcriptional and post-transcriptional levels to regulate gene expression and can alleviate the damage caused by stress on plant growth and development [40]. At present, miRNA screening studies have made important progress in several aspects, including gray mold of tree peony [41], high-temperature response [42], formation of polychromatic flowers [43], formation of lateral branches [44], tree peony flower development [45], seed fatty acid synthesis [46], bud dormancy [47], copper stress [48], and spot color formation [49]. The screening and identification of reference miRNAs for qRT-PCR provided technical support for the development of molecular biology studies of tree peony [50, 51]. Studies have shown that the *PsmiR172b-PsTOE3* module plays a dual role in releasing dormancy and flowering of tree peony [52]. In addition, the *PsmiR159b-PsMYB65* module can release the dormancy of tree peony buds by influencing the cell cycle of tree peony [20–22]. However, there were no reports related to the screening and identification of miRNAs responding to drought stress in tree peony. Among the large number of miRNAs currently known, miR172 is an important member and highly conserved in plants [53]. The available reports on miR172 show that it plays an important role in regulating plant vegetative phase transition [54], organ development [20–22], plant flowering [55], and stress response [56, 57].

To explore the molecular regulation mechanism of tree peony on drought stress, different degrees of drought stress and rehydration treatment to *P. ostii* ‘Fengdan’ were applied, and physiological, transcriptomic, miRNA and degradome analysis methods were employed. miRNAs and their target genes in response to drought stress were screened, and miRNA–mRNA–TF regulatory networks were identified. The expression of miRNA and target genes under drought stress was verified with real-time fluorescence quantitative PCR. Furthermore, overexpression and transient silencing vectors were constructed for genetic transformation. To better assess the effects of drought stress on plants, we selected tobacco—a traditional model plant with a large leaf area. Moreover, to minimize the influence of interspecific variation, we further utilized tree peony leaves and petals as conversion materials in the instantaneous conversion experiments. This strategy provided stronger evidence for our argument compared to the use of traditional model plants. Our studies provide a theoretical basis and technical support for further research and development of new varieties of tree peony with stronger drought resistance.

## Results

### Drought stress resulted in changes in phenotypes, including stomata in *P. ostii* ‘Fengdan’

**Figure 1:**
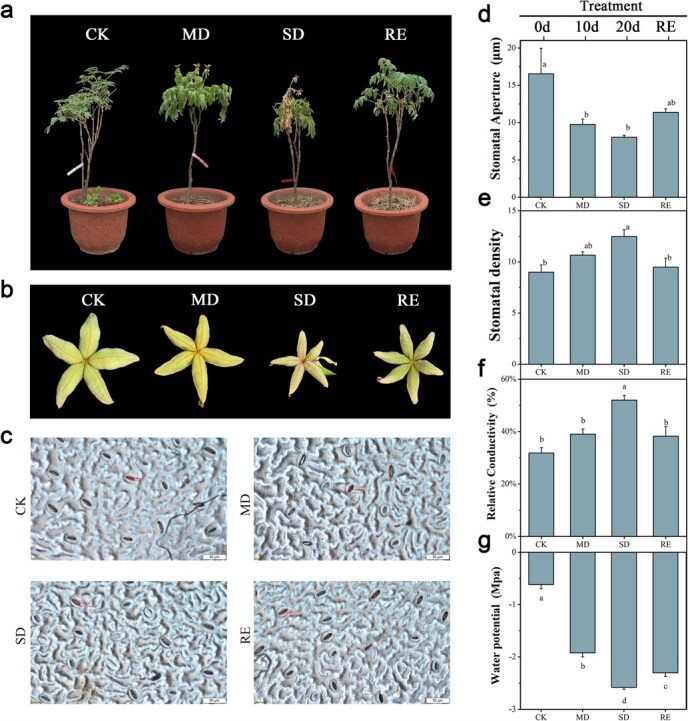
Effects of drought stress on phenotypes and physiology of *P. ostii* ‘Fengdan’. (a) Plant phenotypes after drought treatment. Control Check (CK): *P. ostii* ‘Fengdan’ that were not subjected to drought stress treatment during the initial stage of the experiment on the 0th day; Moderate drought (MD): From 0 to 10 days, 500 ml of water was applied daily to achieve a relative moisture content of 35%; Severe drought (SD): From 10 to 20 days, 250 ml of water was applied daily to achieve a relative moisture content of 25%; Rehydration (RE): From 20d to 30d, apply sufficient watering to restore the plant to its original physiological state. (b) Fruit pod phenotype of *P. ostii* ‘Fengdan’ plants under drought stress. (c) Stomata phenotype of *P. ostii* ‘Fengdan’ plants under drought stress. (d) Stomatal opening of *P. ostii* ‘Fengdan’ plants under drought stress. (e) Stomatal density of *P. ostii* ‘Fengdan’ plants under drought stress. (f) Relative electrical conductivity of *P. ostii* ‘Fengdan’ plants under drought stress. (g) Leaf water potential of *P. ostii* ‘Fengdan’ plants under drought stress. Different letters represent significant differences among lines in Duncan’s multiple range comparison tests: *P* < 0.05.

As can be seen from Fig. 1a, plants in the control group developed normally, with green leaves and straight branches. The plants in the moderate drought group withered, curled and sagged obviously after drought stress, and the plants in the severe drought group withered seriously as a whole, showing symptoms of scorched leaves and delayed development of fruit pods (Fig. 1b). After rehydration, leaf drying symptoms were alleviated, and the leaf base was flattened.

Stomatal opening and density of *P. ostii* ‘Fengdan’ leaves changed significantly under drought stress (Fig. 1c). As shown in Fig. 1d, under normal water conditions, stomatal opening in the control group ranged from 12 to 16 μm. On the 10th day of drought stress, stomatal opening decreased significantly, but there was no significant difference among all groups. On the 20th day of drought stress, stomatal opening decreased to about 7 μm. After rehydration, the porosity of the rehydration group recovered. As can be seen from Fig. 1e, with the persistence of drought stress, leaf stomatal density increased. After 10 days of rehydration, the cell stomatal density decreased (Fig. 1e). The results suggest that the transpiration rate decreased due to the slight closure of leaf stomata under drought stress.

### The leaf membrane system of *P. ostii* ‘Fengdan’ was actively regulated in respond to drought stress

As shown in Fig. 1f, under normal growth conditions, the relative electrical conductivity of leaves in the control group was between 25% and 35%. With the extension of drought stress time, the relative electrical conductivity of leaves increased, and the change in the severe drought group was the most obvious. The relative conductivity after rehydration decreased to a normal level, indicating that the plants had a strong recovery ability. The results of variance analysis showed that the relative electrical conductivity of *P. ostii* ‘Fengdan’ leaves of tree peony under severe drought stress was significantly higher than that of other groups, and the cell membrane of mature leaves of tree peony was seriously damaged.

As shown in Fig. 1g, the leaf water potential value of the normal watering control group was about −0.62 MPa. There were significant differences in leaf water potential under different degrees of drought, which decreased to about −2.0 MPa under moderate drought stress. On the 20th day of drought stress, the water potential continued to decline, reaching about −2.5 MPa during severe droughts. The water potential value recovered slightly after rehydration. The results showed that under severe drought stress, the water loss of *P. ostii* ‘Fengdan’ leaves was more serious and the water potential was lower.

### miRNA sequencing revealed the expression of miRNAs in *P. ostii* ‘Fengdan’ under drought stress

**Figure 2:**
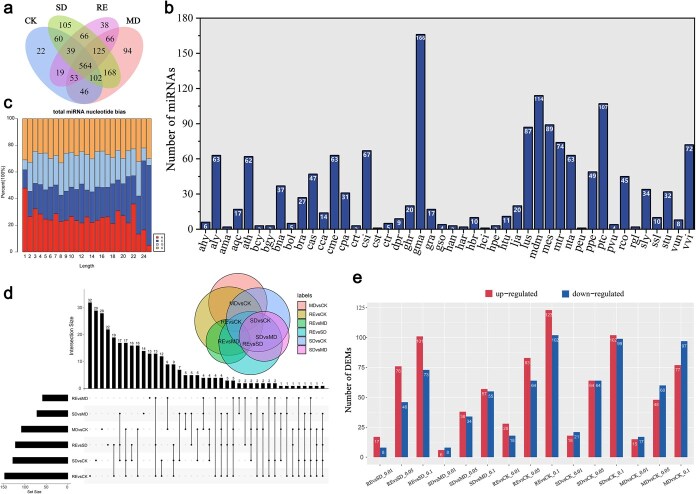
miRNA sequencing revealed the expression of miRNA in *P. ostii* ‘Fengdan’ under drought stress. (a) Number of miRNAs expressed in CK-MD-SD-RE Venn diagram. (b) Conservation of the identified miRNA with other species. (c) Base number analysis of the identified miRNA (d) Distribution of DEMs under drought stress. (e) Number of differentially expressed miRNAs.

As shown in Table S1, a total of 12 sRNA libraries were constructed from CK, MD, SD, and RE groups of plants with three replicates for each group. The Pearson correlation coefficient between the samples used for miRNA sequencing is shown in Fig. S1. An overview of cleaning the original sequences was shown in Table S2. The length distribution of total and unique sRNA counts was shown in Fig. S2. The categories of repeats in sRNA were shown in Table S3. Based on the whole genome sequencing results of tree peony [5], a total of 1125 miRNAs were identified in response to drought stress in *P. ostii* ‘Fengdan’, among which 883 miRNAs were significantly differentially expressed. All expressed miRNAs were shown in Table S4. There were 905, 1217, 1229, and 970 miRNAs expressed in CK, MD, SD, and RE, respectively, among which 564 miRNAs were coexpressed in the four treatment groups. The number of miRNAs expressed in each experimental group was shown in the Fig. 2a. The identified miRNAs matched to 46 different species, among which ‘gma’ (*Glycine max*), ‘mdm’ (*Malus domestica*), and ‘ptc’ (*Populus trichocarpa*) were the most abundant (Fig. 2b). The results of miRNA nucleotide analysis showed that bases A and C account for the largest proportion (Fig. 2c).

As shown in Table 1, a total of 1445 pre-miRNAs and 1568 unique miRNAs were identified. The length distribution of miRNAs count was shown in Table S5. The known and predicted miRNAs were shown in Table S6. MiRNAs were divided into four major groups, Gp1, Gp2a, Gp2b, Gp3, and Gp4. Groups Gp1, Gp2a, Gp2b, and Gp3 included four types of known miRNAs, and group Gp4 only included predicted miRNAs. The number of miRNAs in each group is shown in Table 1.

**Table 1:** Summary of known and predicted miRNA

	Groups	Pre-miRNA	Total	Unique miRNA	Total
Known miRNA	Group1	13	391	20	443
	Group2a	53		76	
	Group2b	303		318	
	Group3	22		29	
Predicted miRNA	Group4	1054		1125	
Total		1445		1568	

In order to identify the differentially expressed miRNA (DEM) related to drought response of tree peony, the miRNA expression of *P. ostii* ‘Fengdan’ in four drought treatment groups was analyzed. The results showed that there were much more differentially expressed miRNAs in SD VS CK and MD VS CK (Fig. 2d). Figure 2e shows the number of upregulated and downregulated DEMs in different groups. Then, six intersections of the four experimental groups were selected for cross-analysis.

### Transcriptome sequencing revealed the expression of *P. ostii* ‘Fengdan’ genes under drought stress

Transcriptome sequencing was conducted on the samples to comprehensively analyze gene expression profiles. Figure S3 shows the Pearson correlation between mRNA-sequenced samples. Table S7 shows an overview of the raw reads, valid reads, Q30, and GC content data. After filtering through 641 million raw readings, 592 million valid readings were obtained. All annotated genes are shown in Table S8.

**Figure 3:**
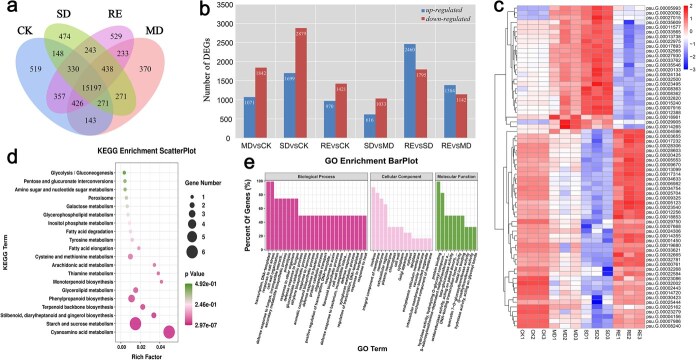
Transcriptome sequencing revealed the expression of *P. ostii* ‘Fengdan’ genes under drought stress. (a) Number of genes expressed in WT-MD-SD-RE Venn diagram. (b) Number of DEGs. (c) Cluster heat map of DEGs expression. (d) KEGG analysis of DEGs. (e) GO analysis of DEGs.

Gene expression under different drought stress levels was analyzed to find differentially expressed genes in response to drought stress. Transcriptome sequencing yielded 35 687 functional genes with an average length of 1296 bp. Among them, 15 786 were differentially expressed. Figure S4 shows an exon model of gene abundance levels normalized to the number of base fragments per kilobyte (FPKM) criteria. The number of genes expressed in each experimental group was shown in Fig. 3a. Figure 3b shows the amount of DEG up and down in different groups. A total of 2913 cross-annotated genes were detected in CK and MD, and 4578 cross-annotated genes were detected in CK and SD. A total of 2391 cross-annotated genes were detected between CK and RE. A total of 1649 cross-annotated genes were detected between MD and SD, 2526 were detected between MD and RE, and 4255 were detected between SD and RE. Compared with CK, the differential genes of MD were upregulated by 1071 and downregulated by 1842, and those of SD were upregulated by 1699 and downregulated by 2879. There were 970 upregulated differential genes and 1420 downregulated differential genes in RE. Compared with SD, 2460 upregulated and 1795 downregulated differential genes were identified in RE. There were 68 common annotated genes among CK, MD, SD, and RE. The expression levels of DEGs varied in different treatment groups. The expression pattern is shown in Fig. 3c.

### GO and KEGG enrichment analysis of differentially expressed genes

Functional enrichment analysis of gene ontology (GO) could determine the main biological functions of differentially expressed genes. Among them, the enrichment results with significant differences were selected as the final results. The significantly enriched categories are shown in Fig. 3e. After hierarchical network and GO enrichment analysis of 68 target genes, 25 biological processes, 15 cell components, and 10 molecular functions were obtained. The two biological processes that accounted for the largest proportion were transcription and DNA-templated and response to salt stress. DEGs were significantly enriched in the process of protein binding, followed by high enrichment in the hydrolase activity, hydrolyzing O-glycosyl compound, and so on.

The metabolic and signal transduction pathways connected to miRNAs and target genes were discovered by the KEGG pathway analysis. Pathways, such as cyanoamino acid metabolism, monoterpenoid biosynthesis, thiamine metabolism, arachidonic acid metabolism, and stilbenoid, diarylheptanoid, and gingerol biosynthesis were identified and are shown in Fig. 3d.

### miRNA–mRNA–TF regulatory networks were identified

By comparing MD vs CK, SD vs CK, RE vs CK, SD vs MD, RE vs MD, and RE vs SD, a total of 28 differentially expressed transcription factors were identified, belonging to 22 transcription factor families. They included five genes in *NAC* families, four genes in *bHLH* families, four genes in *ERF* families, three genes in *FAR1* families, three genes in *MYB* related families, three *genes in NF-YC* families, etc. Among these factors, *NAC* family, *bHLH* family, and *ERF* family account for the largest proportion in order (Table S9).

**Figure 4:**
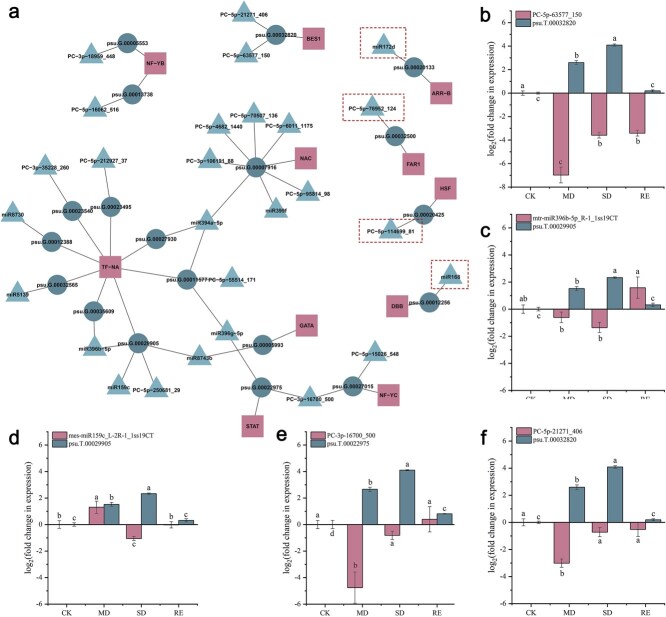
Multi-omics analysis of the regulatory networks of miRNA–mRNA–TF modules. (a) Analysis of the regulatory networks mediated by miRNA–mRNA–TF module identified by drought stress response. (b) QRT-PCR analysis PC-3p-16700_500 and psu.T.00022975. (c) QRT-PCR analysis of mtr-miR396b-5p_R-1_1ss19CT and psu.T.00029905. (d) QRT-PCR analysis of mes-miR159c_L-2R-1_1ss19CT and psu.T.00029905. (e) QRT-PCR analysis of PC-5p-63577_150 and psu.T.00032820. (f) QRT-PCR analysis of PC-5p-21271_406 and psu.T.00032820. Different letters represent significant differences among lines in Duncan’s multiple range comparison tests: *P* < 0.05.

Previous studies have shown that the regulatory relationship between miRNA-target is not always one-to-one. A miRNA can simultaneously regulate multiple mRNAs, and an mRNA can also be synergistically targeted by several miRNAs. Therefore, miRNA target expression does not necessarily correlate negatively. As shown in Fig. 4a, *psu.T.00007916* was simultaneously targeted by seven miRNAs, including *PC-5p-70507_136*, *PC-5p-4682_1440*, and *PC-5p-6011_1175*. *miR8743b* targets both *psu.T.00029905* and *psu.T.00005993* genes. In addition, differences in miRNA and mRNA expression may result from differences in the expression of the biological replication.

To further verify the accuracy of transcriptome data, five pairs of significantly differentially expressed miRNAs and their corresponding target genes were randomly selected for qRT-PCR analysis (*PC-3p-16700_500* and *psu.T.00022975*, *mtr-miR396b-5p_R-1_1ss19CT,* and *psu.T.00029905*, *mes-miR159c_L-2R-1_1ss19CT* and *psu.T.00029905*, *PC-5p-63577_150* and *psu.T.00032820*, *PC-5p-21271_406* and *psu.T.00032820*). As shown in Fig. 4b–f, the expression of miRNAs and their target genes exhibited significant differences under drought stress. Target t-plots (Fig. S5) show the mRNA cleavage sites within target genes silenced by miRNAs during treatments groups, validating the transcriptome sequencing results. Correlation analysis of miRNA and target expression profiles between high-throughput sequencing and qRT-PCR was shown in Fig. S6.

### Target tangent relationship of PomiR172d and PoARR was validated

Among all the combinations presented in Fig. 4a, it was observed that only miR172, miR168, PC-5p-76952_124 and PC-5p-114699_81 exerted their regulatory effects by interacting with their respective target genes and subsequently influencing transcription factors, thereby modulating the physiological mechanisms of tree peony under drought stress. This regulatory pattern is distinct and holds considerable research value when compared to the other miRNAs identified. Based on these findings, miR172 was selected, as an example, for further investigation. It was successfully cloned from tree peony, and transformation studies were conducted to explore its functional role.

**Figure 5:**
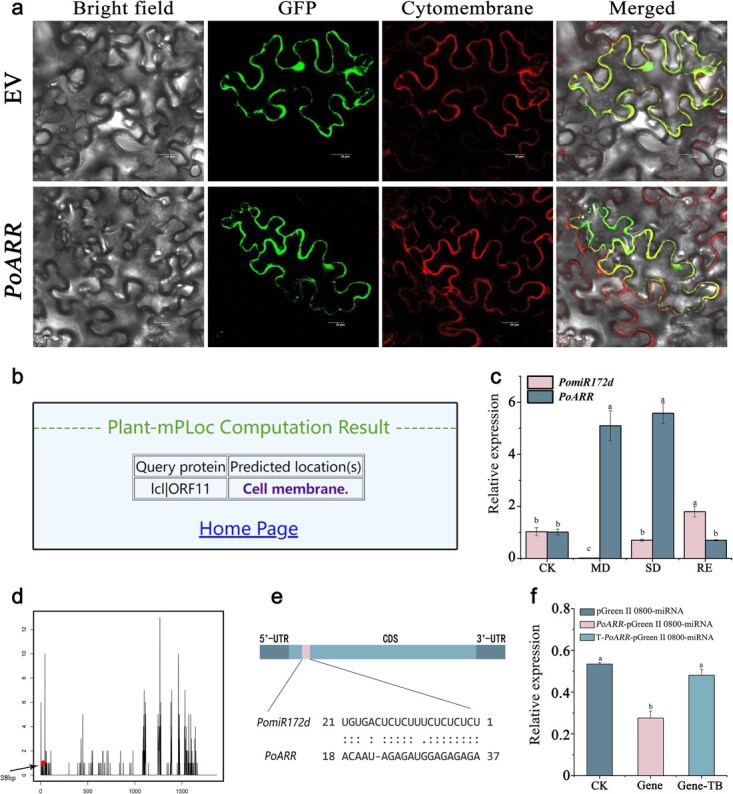
*PomiR172d* targeted regulation of *PoARR*. (a) Subcellular localization of *PoARR* protein. Luminescence was observed under the laser confocal microscope, and its distribution overlapped with that of the cell membrane. EV: empty vector. (b) Prediction of subcellular localization (c) qRT-PCR analysis of *PomiR172d* and *PoARR* under drought stress. (d) Target cut site prediction of degradome. (e) Schematic diagram of *PomiR172d* recognition site on *PoARR* coding sequence. (f) Fluorescence activity analysis of dual luciferase reporter gene assay. Different letters represent significant differences among lines in Duncan's multiple range comparison tests: *P* < 0.05.

Subcellular localization results showed that *PoARR* protein was localized in the cell membrane of tobacco (Fig. 5a), consistent with the predicted results (Fig. 5b).

qRT-PCR expression levels of *PomiR172d* and its target gene *PoARR* in the leaves of *P. ostii* ‘Fengdan’ under drought stress were analyzed, and the results were shown in Fig. 5c. It can be seen that, with the deepening of drought stress, the expression of *PomiR172d* showed a downward trend and was the lowest in moderate drought. The expression of *PoARR* increased significantly and reached the highest level in severe drought, which was moderated after rehydration. It can be seen that *PomiR172d* and the target gene *PoARR* obviously showed a negative regulatory relationship.

The results of multi-omics combined analysis predicted that the cutting site of *PoARR* by *PomiR172d* was at 28 bp of the target gene (Fig. 5d). In order to further verify the targeted cutting relationship between the two, *PomiR172d*-pGreen II 62-SK and *PoARR*-pGreen II 0800-miRNA vectors were constructed and transfected into tobacco leaves by *Agrobacterium*-mediated method. The treated leaves were fully dissolved and the tissue fluid was extracted for further detection. The data were analyzed and processed, and the results were shown in Fig. 5f. It can be seen that *PomiR172d* and *PoARR* group have the lowest activity compared with the other two groups, which is the result of the interaction between *PomiR172d* and *PoARR*, while *PomiR172d* does not affect the empty vector of pGreen II 0800-miRNA and the mutated *PoARR* gene. The relationship of targeted cutting between them is further explained.

### The regulatory effects of PomiR172d–PoARR module on drought stress was validated via transient expression

**Figure 6:**
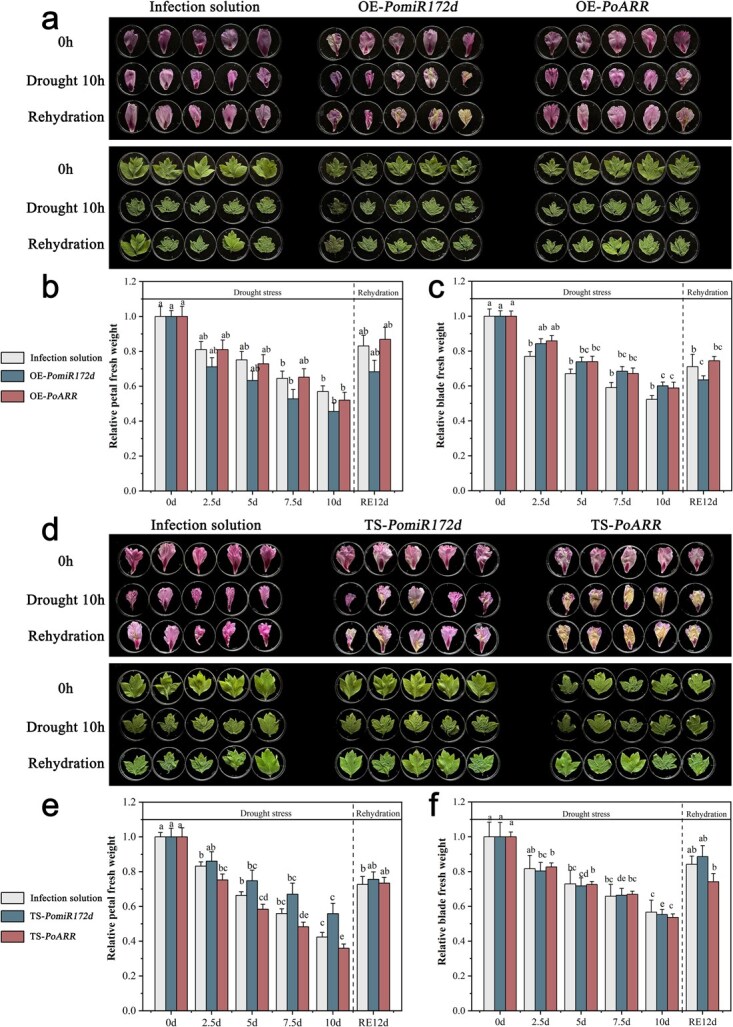
Response of transiently transformed plant materials to drought stress. (a) Transient overexpressed (OE) tree peony petal and leaf responses to drought stress. (b) Transient overexpressed tree peony petal fresh weight changes. (c) Transient overexpressed tree peony leaf fresh weight changes. (d) Transient silent tree peony petal fresh weight changes. (e) Transient silent (TS) tree peony petal fresh weight changes. (f) Transient silent tree peony leaf fresh weight changes. Different letters represent significant differences among lines in Duncan’s multiple range comparison tests: *P* < 0.05.

The petals and leaves of tree peony ‘Luoyang Hong’ were treated with transient overexpression. After overexpression (OE) of *PomiR172d*, the petals and leaves were wilted obviously, and the whole petals showed whitening, which was not alleviated after rehydration. However, the state of petals and leaves of OE-*PoARR* was significantly better than that of OE-*PomiR172d*, and the petals did not show obvious fading, only slightly wilting (Fig. 6a). The fresh weight analysis results of petals and leaves were shown in Fig. 6b and c. At the 12th hour of drought, the fresh petals sample quality of OE-*PomiR172d* was only about 40% of the original quantity, while that of OE-*PoARR* was about 85% of the original level, and the change range was significantly lower than that of OE-*PomiR172d*. In general, it was obvious that the indexes of petals and leaves of OE-*PoARR* changed less than those of the other three groups.

*PoARR* and *PomiR172d* were transiently silenced (TS) individually in leaves of tree peony ‘Luoyang Hong’. Compared with TS-*PoARR*, plant materials of TS-*PomiR172d* were less wilted and did not fade obviously. However, after silencing *PoARR*, plant materials showed a dry and yellow state, and the symptoms of drought damage were serious (Fig. 6d). The fresh weight index of treated petals changed significantly, with silencing *PoARR* dropping to about 35% of the original fresh weight at the 10th hour of drought, while TS-*PomiR172d* accounting for about 50% of the original fresh weight. The overall trend of fresh weight of leaves was similar to that of petals, both of which increased significantly after rehydration (Fig. 6e and f).

### Stable transformation of tobacco with PomiR172d and PoARR reveals opposite effects during drought stress

**Figure 7:**
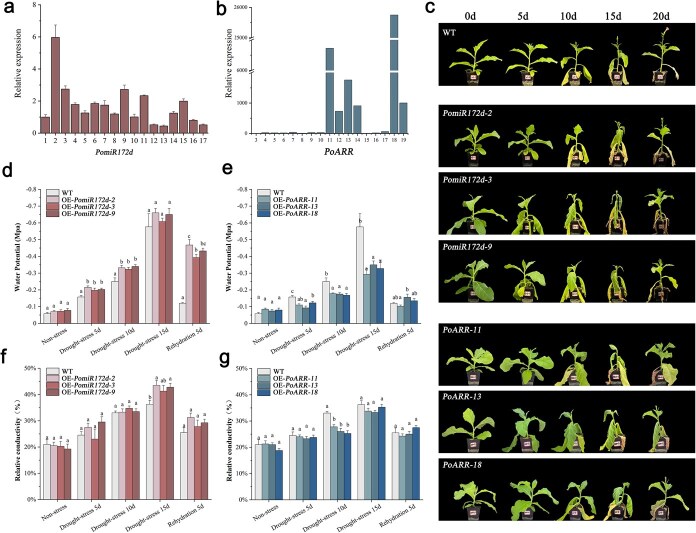
Effects of drought stress on transgenic tobacco. (a) qRT-PCR analysis of OE-*PomiR172d* plants. (b) qRT-PCR analysis of OE-*PoARR* plants. (c) Phenotypic changes of transgenic tobacco plants under drought stress (d) Changes in relative conductivity of OE-*PomiR172d* transgenic plants under drought stress (e) changes in water potential of OE-*PomiR172d* transgenic plants under drought stress (f) Changes in relative conductivity of OE-*PoARR* transgenic plants under drought stress (g) Changes in water potential of OE-*PoARR* transgenic plants under drought stress. Different letters represent significant differences among lines in Duncan’s multiple range comparison tests: *P* < 0.05.

The expression levels of *PomiR172d* and *PoARR* in leaves of transgenic tobacco plants were analyzed in Fig. 7a and b. Accordingly, we selected the three plant lines exhibiting the highest overexpression levels for subsequent experiments.

Phenotypic changes of OE-*PomiR172d* and OE-*PoARR* transgenic tobacco plants under drought stress are shown in Fig. 7c. The leaves of wild-type tobacco plants were wilted under natural drought, and the leaves were obviously yellow after drought stress. The wilting was the most serious on the 15th day of drought and eased after 5 days of rehydration. The plants of OE-*PomiR172d* had more severe drought symptoms than the wild type, and the overall growth of the plants slowed down. Compared with the abovementioned two groups, the leaves of OE-*PoARR* plants showed normal green from 0 to 10 days and did not appear obvious yellow state, and the leaves only slightly wilted, and the plants also returned to normal growth after 5 days of rehydration. In summary, compared with the wild type, the drought resistance of OE-*PomiR172d* plants was weakened, while the drought resistance of plants OE-*PoARR* was enhanced.

The results of water potential determination of OE-*PomiR172d* and OE-*PoARR* transgenic tobacco leaves under drought stress are shown in Fig. 7d and e. The normal leaf water potential of tobacco was about −0.05 Mpa, and the leaf water potential of each group showed a downward trend after drought stress. On the 5th to 15th day of drought stress, the difference between wild type and transgenic gradually appeared, and the difference was most significant on the 15th day of stress, when the water potential of wild-type leaves was about −0.6 Mpa, and that of OE-*PomiR172d* plants dropped to −0.6 to −0.7 Mpa, while that of OE-*PoARR* was about −0.3 Mpa. After 5 days of rehydration, the difference was extremely significant, and the plants water potential of wild-type and OE-*PoARR* significantly recovered, but the water potential of OE-*PomiR172d* transgenic tobacco leaves did not significantly recover.

The relative electrical conductivity of OE-*PomiR172d* and OE-*PoARR* transgenic tobacco under drought stress was shown in Fig. 7f and g. Under normal conditions, the relative electrical conductivity of tobacco plants was about 20%. Under drought stress, the relative electrical conductivity of leaves showed a gradual increasing trend. From Day 0 to Day 10 of drought stress, the relative conductivity of OE-*PoARR* plants increased to about 25%. At the 15th day, the relative conductivity of OE-*PoARR* plants was about 33%, that of wild-type plants was about 35%, and that of OE-*PomiR172d* transgenic tobacco had increased to more than 40%. After rehydration for 5 days, the recovery level of OE-*PomiR172d* transgenic tobacco was lower than the other two.

**Figure 8:**
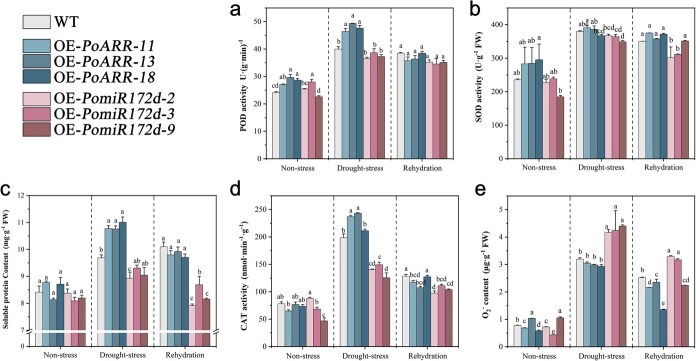
Determination of activity indexes of ROS-scavenging enzymes in overexpressed plants under drought stress. (a) Determination of POD activity in overexpressed plants under drought stress. (b) Determination of SOD activity in overexpressed plants under drought stress. (c) Determination of soluble protein content in overexpressed plants under drought stress. (d) Determination of CAT activity in overexpressed plants under drought stress. (e) Determination of O^2−^ content in overexpressed plants under drought stress. Different letters represent significant differences among lines in Duncan's multiple range comparison tests: *P* < 0.05.

The activity of enzymes scavenging reactive oxygen species (ROS) in transgenic tobacco plants of OE-*PomiR172d* and OE-*PoARR* was studied under drought stress, and the results were shown in Fig. 8. The peroxidase (POD) activity detection results showed that the POD activity of the overexpressed plants and the wild type both showed an upward trend under drought stress (Fig. 8a). And the activity of OE-*PomiR172d* plants was slightly lower than that of wild type, while the index of OE-*PoARR* plants was significantly higher than that of the other two groups. POD activity decreased after rehydration. The results of superoxide dismutase (SOD) activity detection showed that OE-*PomiR172d* plants were lower than those of wild type and OE-*PoARR* in the drought period, but the overall variation among the three plant groups was small. OE-*PoARR* and OE-*PomiR172d* showed differences between these two groups after rehydration (Fig. 8b). The results of soluble protein content and catalase (CAT) activity were similar to the above, and the results of OE-*PoARR* plants were significantly higher than those of wild type plants and OE-*PomiR172d* plants in the drought stress stage, and the soluble protein content also showed a significant difference after rehydration treatment (Fig. 8c-d). The detection results of O^2−^ content were shown in Fig. 8e. It can be seen that wild type and OE-*PomiR172d* plants increased significantly under drought stress, and the O^2−^ content was about twice as high as that under normal growing conditions, while the increase rate of OE-*PoARR* plants was slight. O^2−^ content reached its peak in all groups under drought stress, especially in plants of OE-*PomiR172d*. After rehydration, O^2−^ content in all three groups decreased.

In summary, the overexpression of *PoARR* resulted in an increase in antioxidant enzyme activity in tobacco plants, reducing ROS levels and significantly improving drought tolerance. Conversely, the drought resistance of plants after overexpression of *PomiR172d* was weak, and the conclusions further verified the negative correlation between the two.

## Discussion

Drought stress is one of the most harmful factors to plant development. Enhancing crop stress resistance and breeding new varieties with high yield and resistance are currently the most economical ways to increase agricultural productivity [58, 59]. A combined multi-omics analysis can correlate information obtained at different levels of an organism and help reveal the complex mechanisms of plant response to stress at a holistic level [60, 61]. For the past few years, a great deal of studies has shown the physiological and ecological response of tree peony to drought stress [6, 28, 30, 34, 62], but the understanding of the molecular mechanism of tree peony in response to drought stress is still limited. The effects of different levels of drought stress on physiological and biochemical indexes of *P. ostii* ‘Fengdan’ were studied in this study. Combined miRNA, transcriptome, and degradome of leaves of *P. ostii* ‘Fengdan’ under drought stress were analyzed to further the understanding of the molecular mechanism underlying tree peony's response to drought stress.

The obvious inhibition of plant development is the most intuitive response of plants to drought stress [63]. When plants are exposed to drought stress, a series of physiological responses is triggered [58, 64]. In this study, the leaves’ relative electrical conductivity of tree peony increased noticeably with water loss and biofilm damage, the water potential decreased significantly and the water potential of leaves in severe drought decreased by nearly four times compared with before. With the deepening of drought stress, *P. ostii* ‘Fengdan’ leaves showed significant changes in morphology, stomatal opening, and stomatal density in this study, substantiating the results of a study on *Berberis poiretii* leaves treated with drought stress [65]. This previous study indicated that the *Berberis* leaves reduced the stress damage by reducing stomatal opening and reducing transpiration.

miR172, which is widely present in plants and was first cloned in *Arabidopsis thaliana*, is believed to play a fundamental role in plant growth and development [66]. In this study, we found that the miR172 family mainly existed in angiosperms and was distributed in 46 species of 17 families and 3 classes. Most of these angiosperms are magnolia species, while monocotyledonous species are relatively in small numbers. In this study, the degradome sequencing predicted that the target gene of *PomiR172d* was *PoARR*, and the functional annotation results showed that the gene was involved in the biological process of transmembrane transport, and the TF annotation information showed that the gene was a member of the transcription factor ARR family. It was speculated that *PoARR* could regulate the transmembrane transport process of tree peony plants in response to their own regulation and enhance the resistance of tree peony plants to drought environments when they were under drought stress. In this study, real-time fluorescence quantitative PCR analysis showed that with the deepening of drought stress, *PomiR172d* expression showed a downward trend, and it was the lowest in moderate drought. The expression of *PoARR* increased significantly and reached the highest level in severe drought, which was moderated after rehydration. Thus, *PomiR172d* and the target gene *PoARR* obviously showed a negative regulatory relationship. These results indicated that *PomiR172d* and *PoARR* were active in response to drought stress and regulated the resistance of tree peony plants to drought stress. When *PomiR172d* and *PoARR* were coexpressed together, luciferase had the lowest activity, while *PomiR172d* did not act on the empty vector of pGreen II 0800-miRNA and the mutated *PoARR* gene, verifying the targeted cutting relationship between the two groups.

Han et al. concluded that miR172 in *Arabidopsis* was related to drought stress through overexpression genetic transformation [67]. This also indicates that the analysis results of the correlation between miR172 and drought stress identified by us are consistent with previous studies. Phenotypic and functional analysis further confirmed the important functions of *PomiR172d* and its target gene *PoARR* in response to stress. After drought treatment of transgenic plants, it can be seen that with the extension of drought time, *PomiR172d* plants overexpressing *PomiR172d* had more serious symptoms of drought damage than the wild type, the leaves wilted and yellow, and the overall growth of the plants was poor. The plant materials that overexpressed *PoARR* were in a better condition. After the physiological indexes of transgenic plants were determined, combined with the analysis results of membrane permeability of transgenic plants, it could be seen that *PomiR172d* overexpression reduced the drought resistance ability of tobacco. This is consistent with the results of Zhou et al. 's study on gma-miR398c, which found that the expression level of target genes in soybeans overexpressed with gma-miR398c was decreased, and the drought tolerance of soybeans was reduced [68].

**Figure 9:**
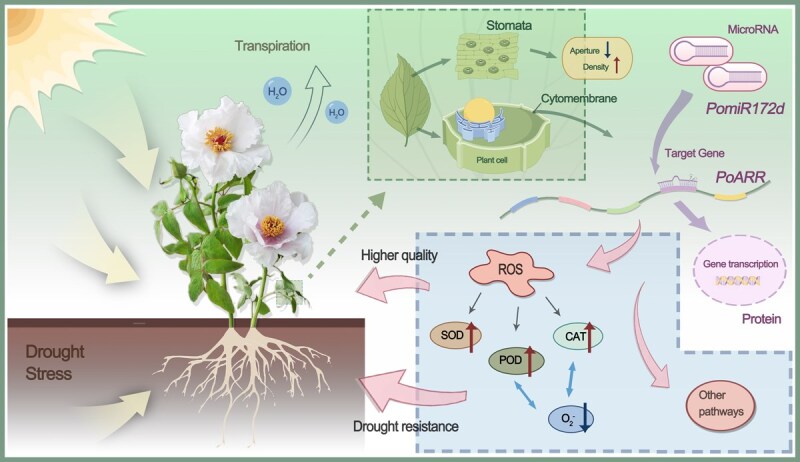
Model diagram of *PomiR172d* targeting *PoARR* to influence drought resistance in *P. ostii* ‘Fengdan’ through the ROS pathway.

Antioxidant enzymes SOD, POD, and CAT can effectively remove ROS in plants. In this study, by analyzing the enzyme activities in transgenic plants, including POD, SOD and CAT, we found that the overexpression of *PoARR* can enhance the activity of antioxidant enzymes and reduce the accumulation of ROS, leading to enhanced drought resistance of plants (Fig. 9). The result of Wang et al. showed that *PomiR396g-5p* regulated plant ROS levels by inhibiting the expression of target gene *PoACO1*, thus affecting the response of tree peony to drought stress [29].

These findings demonstrate that the expression of *PomiR172d* is inversely correlated with that of the *PoARR* gene. Furthermore, plants with silenced *PoARR* exhibit increased sensitivity to drought stress. Collectively, these data support the hypothesis that *PoARR* positively regulates drought tolerance in *P. ostii* ‘Fengdan’ by enhancing the activity of the ROS scavenging system. In the study of *miR166* by Li et al., inhibition of miR166 expression by Short tandem target mimic (STTM) technology significantly improved the abiotic stress resistance of maize, especially drought tolerance, which was consistent with the results of this study (Li et al., 2020). An et al.'s study on the *PoVQ31* gene demonstrates that plants with *PoVQ31* gene silencing exhibit increased sensitivity to drought [69]. The authors suggest that *PoVQ31* may enhance drought tolerance in *P. ostii* ‘Fengdan’ by activating the ROS scavenging system. This hypothesis is in line with our preliminary findings. Our study has established a negative correlation between *PomiR172d* and *PoARR*, as well as their involvement in the ROS pathway in tree peony. Nevertheless, the precise molecular mechanisms through which *PomiR172d* and *PoARR* regulate ROS enzyme activities remain to be elucidated in future investigations. These findings will provide a valuable foundation for further enhancing drought tolerance in tree peony.

## Conclusion

In this study, through comprehensive analyses of transcriptomics, miRNA and degradation omics, 19 differentially expressed miRNAs were successfully identified, which could regulate 189 differentially expressed genes, and the expression regulatory network between miRNAs and their target genes was constructed. Drought response miRNA–mRNA–TF modules, such as *miR172d-psu.T.00020133.1-ARR* were identified. The cutting cleavage of target gene mRNAs by plant miRNAs is usually a major mode of regulating target genes. Therefore, the results of this study further verified this negative correlation between the expression of *PomiR172d* and its target gene *PoARR*, and also confirmed that overexpression of the target gene *PoARR* increased plant ROS scavenging for homeostasis and thus enhanced the resistance of tree peony to drought stress.

## Materials and methods

### Treatment of plant materials and determination of physiological indexes

Nine-year-old plants of the *P. ostii* ‘Fengdan’ cultivar that had uniform growth and were disease-free were selected from the field and planted in pots with an inner diameter of 51 cm and a height of 40 cm, with 1 plant per pot. Normal fertilizer and water maintenance was initially administered to ensure healthy growth. According to Zhang's method, drought stress was artificially simulated by controlling pot moisture [70]. The relative water content of soil in each treatment group was determined through quantitative watering tests based on the soil moisture content of the experimental farmland as the standard. Soil water content was measured by drying the soil in an oven at 100°C to 105°C for 12 hours, and the relative water content of the soil was calculated as a percentage.

In the process of drought stress, samples were taken every 10 days at 9:00 a.m., and one pot of plants was selected from each treatment group for photo recording. When sampling, fully unfolded leaves from the top to the third or the fourth leaf were selected, part of which was used for the measurement of electrical conductivity, water potential, and porosity with each measurement having three replicates. Another portion of the leaves was frozen with liquid nitrogen and stored at −80°C for subsequent determination of physiological indexes.

Leaf water potential was measured by a PSYPRO (ELITechGroup, Logan, USA), relative conductivity was measured by a DDS-11A conductivity meter (Instrumentation analysis, Shanghai, China), stomatal opening and density were measured using an inverted fluorescence microscopy (DMi8, Leica, Wetzlar, Germany). According to Li's method, superoxide dismutase (SOD) activity was determined by the nitrogen blue tetrazole method, peroxidase (POD) activity was determined by guaiacol oxidation method, superoxide anion (O^2−^) content was determined by the hydroxylamine method, and catalase (CAT) content was determined by the enzyme lysis method, oluble protein content was determined by Coomassie blue staining [71].

### RNA extraction, library construction, and transcriptome sequencing

Total RNAs were extracted using a SteadyPure Universal RNA Extraction Kit (AG, Hunan, China), and miRNAs were extracted using an RNAiso for Small RNA Kit (TaKaRa, Beijing, China). RNA amount and purity quantification were performed on a NanoDrop ND-1000 (NanoDrop, Wilmington, DE, USA). A transcriptome library was constructed using the NEB Next Ultra RNA Library Prep Kit for Illumina (NEB, USA), a miRNA library was constructed using the TruSeq Small RNA Sample Prep Kits (Illumina, San Diego, USA). Transcriptome sequencing was performed by the 2 × 150 bp paired-end sequencing (PE150) on an Illumina Novaseq™ 6000 (LC-Bio Technology CO., Ltd, Hangzhou, China). miRNAome and degradome sequencing were performed by the 1 × 50 bp single-end approach on an Illumina Hiseq2500.

### Cloning and subcellular localization of PomiR172d and PoARR

Using cDNA as the template, DNA fragments containing *PomiR172d* precursor (*Pre-PomiR172d*) and complete *PoARR* fragment were cloned, respectively. Primers are shown in Table 2. The recombinant plasmid pCAMBIA1304-*PoARR*-GFP was transformed into *Agrobacterium* GV3101, which was then used to infect leaves of *Nicotiana benthamiana* L. by injection. *Agrobacterium PoARR* bacteriological solution, no-load bacteriological solution and membrane Marker *Agrobacterium* bacteriological solution were mixed in proportion during injection. After infection by injection and low light culture for 2 to 3 days, the leaves of the infected site were cut and the fluorescence position was observed by laser confocal microscopy (OLYMPUS, Tokyo, Japan).

**Table 2:** Primer sequences

Function	Name	Sequences(5′-3′)
Cloning	P-*PoARR*-F	ATGGACAGAGAAGATCAAAAGC
	P-*PoARR*-R	GGACACTGTATCTATAGGGATTATAG
Vector construction	NC- *PomiR172d* -F	agtggtctctgtccagtcctTCACACTGTCTGCCCTGTCTC
	NC- *PomiR172d*-R	ggtctcagcagaccacaagtGACATAACAGAGAAGCCCACC
	NC-*PoARR*-f	agtggtctctgtccagtcctATGGACAGAGAAGATCAAAAGC
	NC-*PoARR*-r	ggtctcagcagaccacaagtGGACACTGTATCTATAGGGATTATAG
qRT-PCR	Q-*PoARR*-F	GGGTCCTGGTGCTCAAATCA
	Q-*PoARR*-R	CAGACCATTACAGCGGAGGG
	Q-*PomiR172d*	CAGCGGCACCTCTCTTTCTCTCA
Double luciferase reporter gene assay	62SK-*PomiR172d*-F	cgcggtggcggccgctctagaTCTCTCTCTTTCTCTCAGTGTGAACAC
	62SK- *PomiR172d*-R	ttcctgcagcccgggggatccTGTTTTCTGTAAAAGGTATAATTGCACT
	0800-*PoARR*-F	agatcgccgtgtaattctagaAGCAGAAGGAAAGGAAAATGGA
	0800-*PoARR*-R	agcgaattcactagtggatccCAAGCTGAAACCAGTCACAGATGC
VIGS	TRV2-*PoARR*-F	agaaggcctccatggggatccGGAAAGGGACTCTGGTTGGG
	TRV2-*PoARR*-R	gagacgcgtgagctcggtaccAACTCATAGTTGACTATGTAAGTTAAAGAATCA

In the primer sequence, uppercase letters denote the nucleotide bases, whereas lowercase letters represent enzyme recognition sites or protective bases.

### Verification of target tangent relationship between PomiR172d and PoARR

pGreenII plant expression vectors pGreenII 62-SK and pGreenII 0800 were used to detect the relationship of targeted cutting between *PomiR172d* and *PoARR*. The *PomiR172d* clone was attached to the pGreenII 62-SK vector, and the CDS sequence of *PoARR* was reassembled into the pGreenII0800-luciferase (LUC) plasmid. Primers are shown in Table 2. In order to study the effect of *PomiR172d* on *PoARR*, the mutated *PoARR* sequence was recombined into pGreenII0800-luciferase (LUC) plasmid as a negative control. The pGreenII 0800 vector was used as a blank control.

All recombinant plasmids and empty vectors were transformed into *Agrobacterium* LBA4404 containing pSoup plasmids, and then tobacco infection was carried out according to the instructions of Dual Luciferase Reporter Gene Assay Kit (Yeasen, Shangai, China). The kit was used for fluorescence detection using the GloMax Navigator microplate luminescence instrument (Promega, Beijing, China).

### Analysis of transient overexpression and silenced expression in tree peony by *Agrobacterium tumefaciens*–mediated transformation

The recombinant plasmids pCAMBIA1304-*PomiR172d* and pCAMBIA1304-*PoARR* were transformed into *Agrobacterium* GV3101 and transformed into the petals and leaves of tree peony ‘Luoyang Hong’. After infection, they were cultured at 8°C for 1 to 2 days and at 23°C for 1 day. After the treatment, the surface moisture was sucked up with filter paper and cultured at room temperature. The length, width and fresh weight of petals and leaves were measured at 0, 3, 6, 9, and 12 hours after rehydration, respectively. The samples were quickly frozen with liquid nitrogen and stored in at −80°C for follow-up tests.

The STTM approach was used to knock out *PomiR172d* and construct STTM-*PomiR172d* expression vector. TRV2-*PoARR* vector based on virus-induced gene silencing (VIGS) was constructed. Primers are shown in Table 2. After the construction was completed, *Agrobacterium* GV3101 carrying the construct was used to infect the petals and leaves of tree peony ‘Luoyang Hong’. Drought stress treatment was carried out and physiological indexes were determined using the same method as in Drought stress resulted in changes in phenotypes, including stomata in *P. ostii* ‘Fengdan’ section.

### Evaluation of tobacco transgenic plants and their drought resistance

The recombinant plasmids pCAMBIA1304-*PomiR172d* and pCAMBIA1304-*PoARR* were transferred into *Agrobacterium* GV3101. *SR1* tobacco (*Nicotiana tabacum* cv. Petit Havana SR1) was transformed by the leaf disk method. After pre-culture, coculture, differentiation, and root culture, the final resistant plant was obtained. The three transgenic lines and wild type tobacco were transferred to a culture room and watered regularly to ensure their normal growth. After 1 to 2 weeks, natural drought treatment (no watering) was adopted, and after 15 days of drought, rewater treatment was carried out for 5 days. The experiment was photographed and recorded every 5 days, and the water potential and relative electrical conductivity of transgenic tobacco leaves were measured with three replicates. At the same time, the leaf samples were harvested with liquid nitrogen and placed in a freezer at −80°C for subsequent tests.

## Supplementary Material

Web_Material_uhaf252
