# Supplementary figures and images for: PomiR172d-PoARR module regulates the drought response through the reactive oxygen pathway in tree peony
Source: Hortic Res. 2025 Sep 17;13(1):uhaf252. doi: 10.1093/hr/uhaf252 (PMC12856503; doi:10.1093/hr/uhaf252)

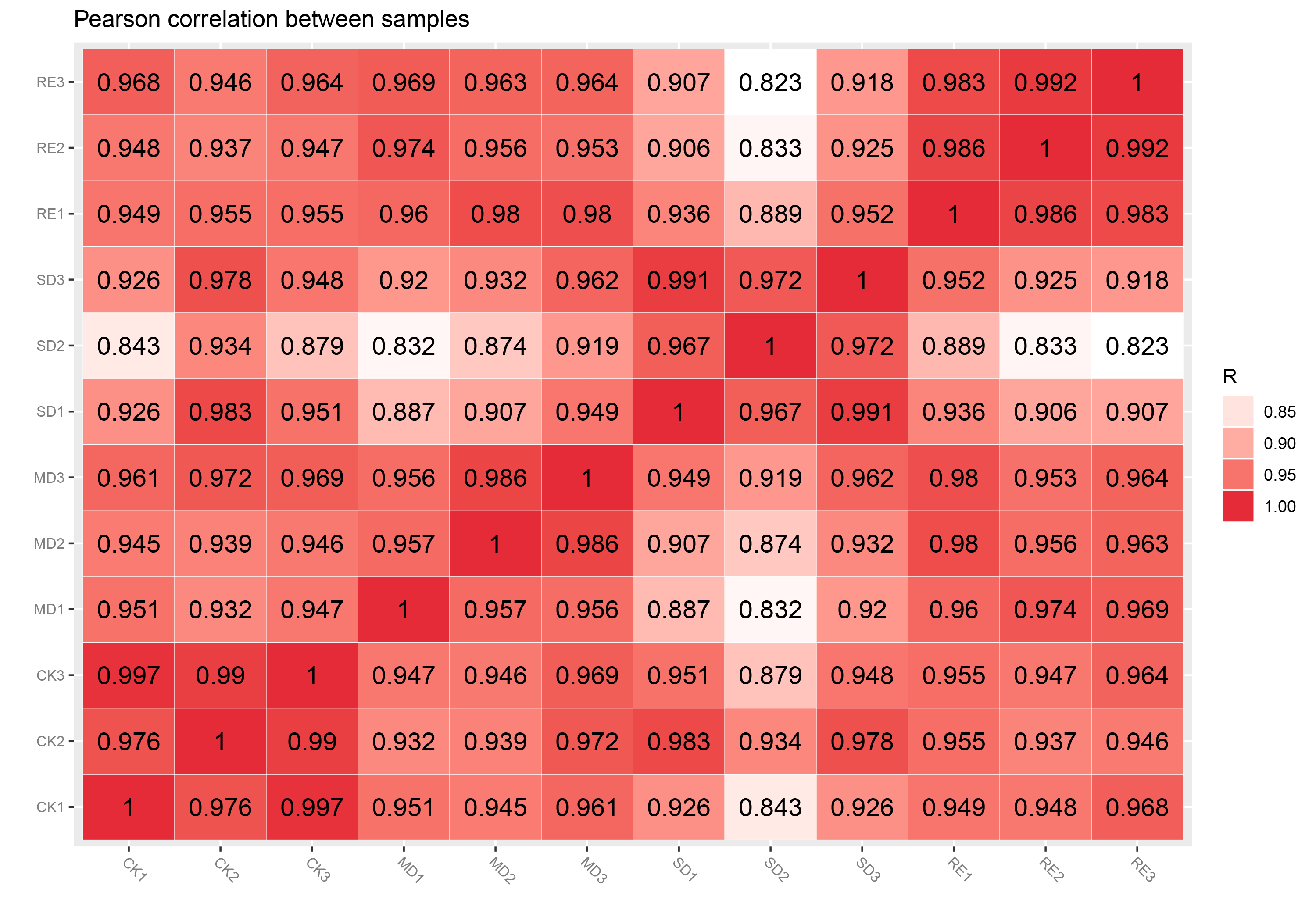

Supplement: Web_Material_uhaf252 [file web_material_uhaf252.zip › Figure S1.tif]

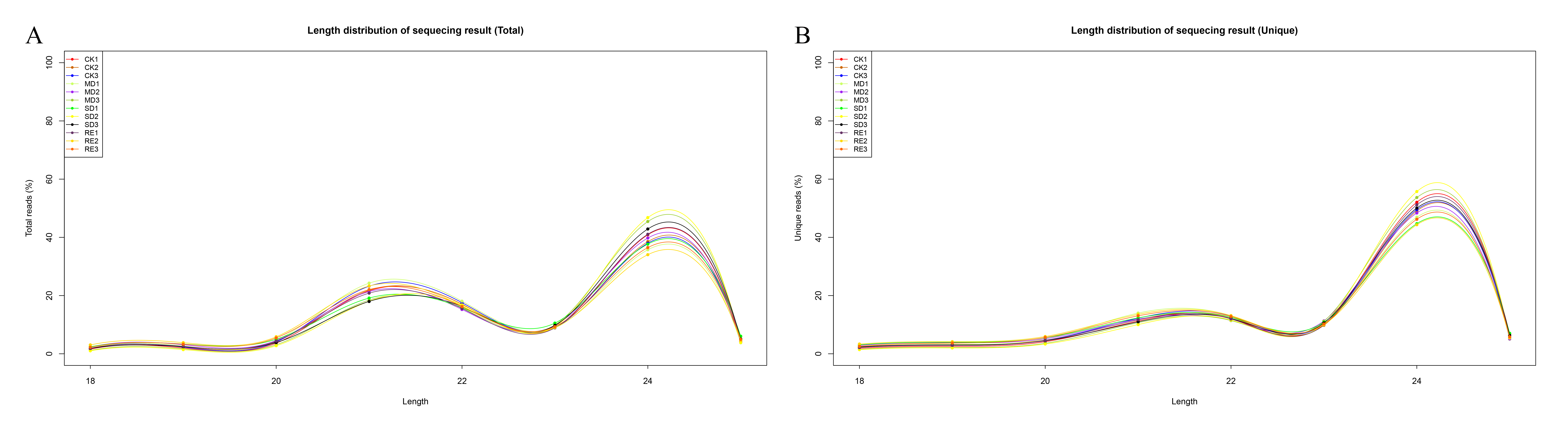

Supplement: Web_Material_uhaf252 [file web_material_uhaf252.zip › Figure S2.tif]

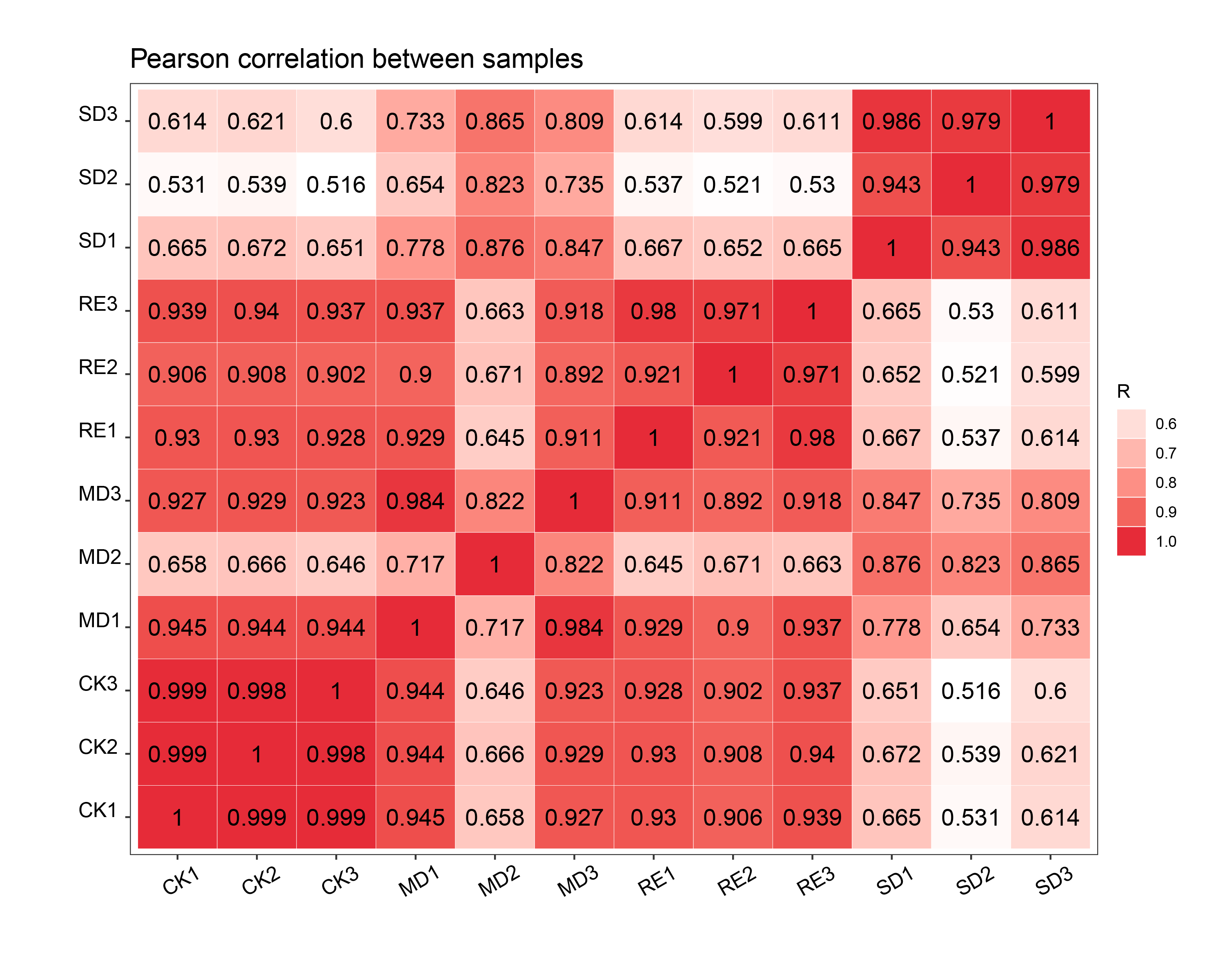

Supplement: Web_Material_uhaf252 [file web_material_uhaf252.zip › Figure S3.tif]

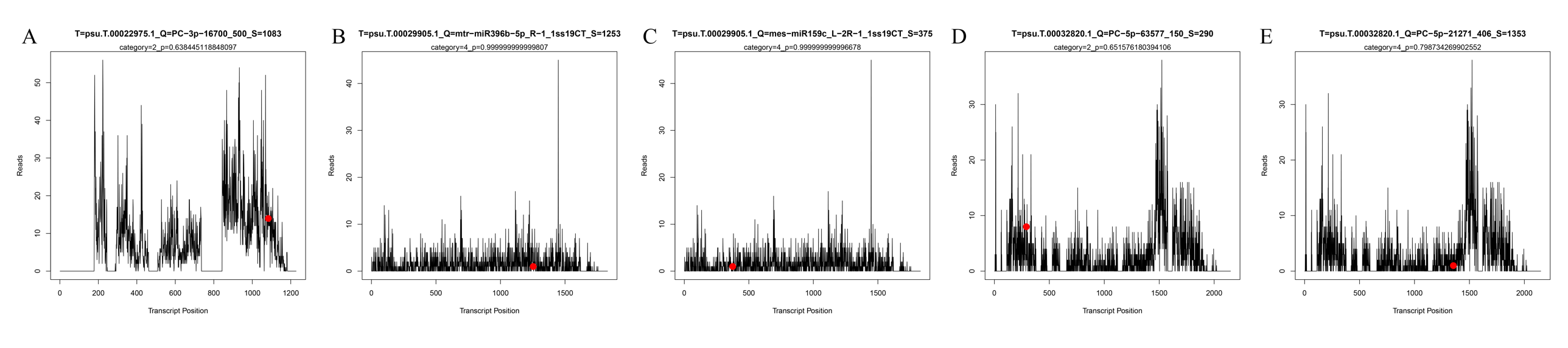

Supplement: Web_Material_uhaf252 [file web_material_uhaf252.zip › Figure S5.tif]

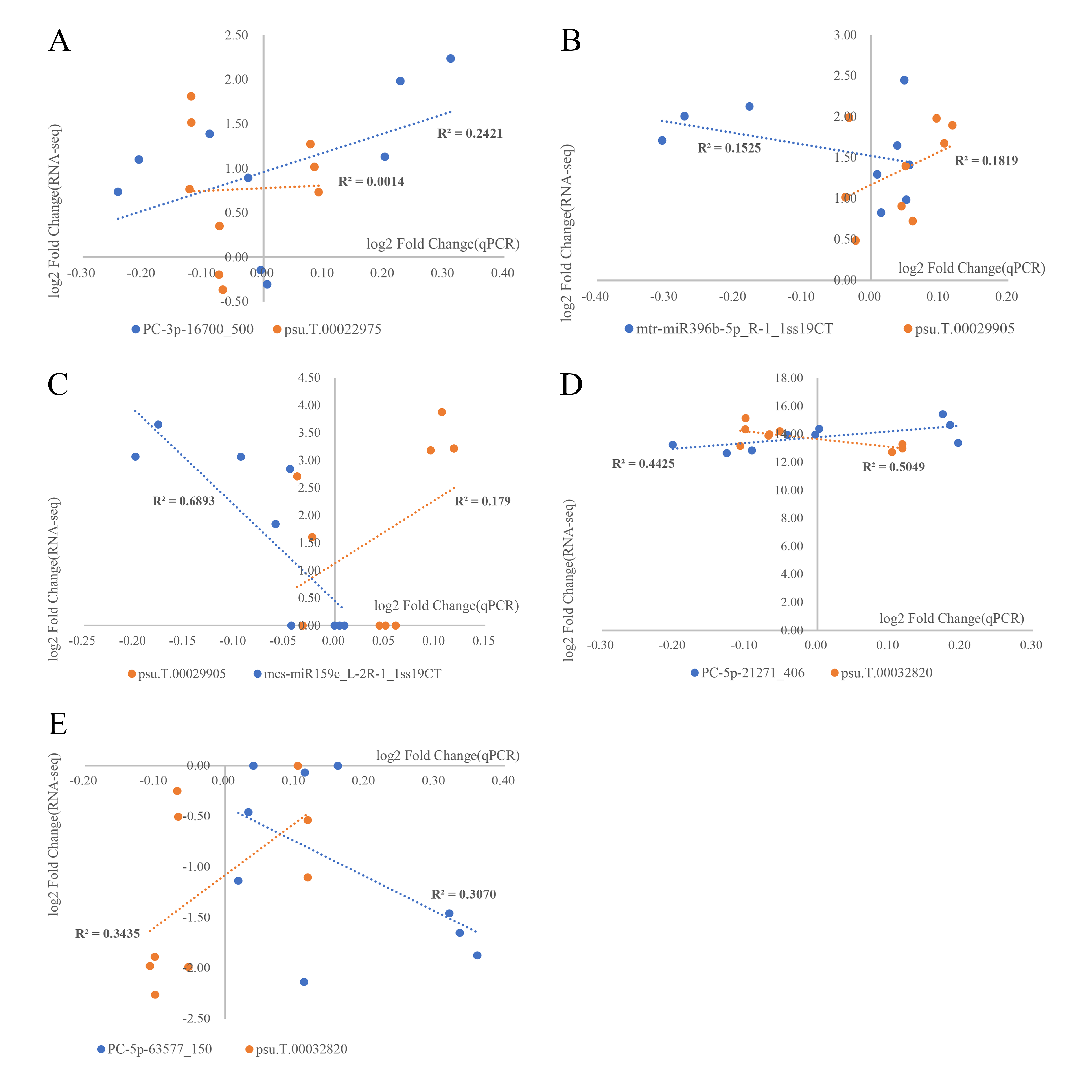

Supplement: Web_Material_uhaf252 [file web_material_uhaf252.zip › Figure S6.tif]
